# Time to Eat Your Vegetables: The Role of Circadian Clocks in Insect Herbivory

**DOI:** 10.3390/insects17020139

**Published:** 2026-01-26

**Authors:** Lena Smith, Connor J. Tyler, Shubhangi Mahajan, Haruko Okamoto, Herman Wijnen

**Affiliations:** 1 School of Biological Sciences and Institute for Life Sciences, University of Southampton, Southampton SO17 1BJ, UK; ls9g20@soton.ac.uk (L.S.); ct13g15@southamptonalumni.ac.uk (C.J.T.); sm6n22@soton.ac.uk (S.M.); h.okamoto@sussex.ac.uk (H.O.); 2 School of Life Sciences, Faculty of Science, Engineering, and Medicine, University of Sussex, Brighton BN1 9RH, UK

**Keywords:** insect herbivory, plant defense, circadian clock, entrainment, output, agriculture, chronoculture, insecticide, ecology, phase alignment, *Drosophila*, *Arabidopsis*, lepidoptera

## Abstract

Insects exhibit a range of ecological relationships with plants, including pollination, seed dispersal, parasitism, predation, and herbivory. Interactions between insects and plants are shaped by internal daily timekeeping systems in both sets of organisms termed circadian clocks. This review describes the impact of the circadian clocks of insects and plants on herbivory, which is highly relevant not only to natural ecosystems, but also to agriculture and forest management. Following an introduction to the circadian clocks of plants and insects, we discuss the circadian organization of relevant aspects of plant metabolism and defense. Next, we describe how insect clocks govern herbivory-associated physiology and behavior before exploring how rhythmic processes in plants and insects interact to temporally control herbivory. Finally, we describe how insights from the clock control of herbivory may inform pest management strategies and what future research in this area may contribute.

## Simple Summary

Many living organisms have internal circadian clocks that not only help them keep in sync with daily rhythms in their environment but also schedule their bodily functions and behaviors. In this review, we look at how such clocks influence when insect feed on plants and how this knowledge could help us manage pests more effectively. We start by discussing insect–plant interactions and the damage that insects cause to crops. Then, we describe the circadian clocks of insects and plants and how the rhythms that they generate shape feeding behavior. Finally, we explore recent research on the timing of insects feeding on plants and discuss how such interventions could lead to better, more sustainable pest control strategies.

## 1. Introduction

### 1.1. Insect–Plant Interactions and Their Ecological Significance

Insects make up roughly 50% of described species and are integral to global ecosystems [1,2]. Their diversification, associated with the radiation of angiosperms during the Cretaceous period [3], enabled them to harness plant resources such as nectar, pollen, and plant tissues for survival [4]. Now, half of all insects are dependent on plant tissue, via egg laying, as a food source or as shelter [5]. In response, plants have evolved a myriad of diverse defenses: chemical and physical, direct and indirect [4,6]. Insect–plant relationships have driven mutualistic coevolution, shaped ecological niches, and fueled an arms race as each organism adapts to overcome improvements to the other’s attack or defense. The mere existence of specialist insects that not only tolerate plant defenses but also harness them as counter defenses or as feeding and/or oviposition cues [7,8] is a testament to how close and complicated the relationships between plants and insects are. Insect–plant relationships have supported the development of ecological niches and entire food networks, with insects acting as primary and secondary consumers and as detritivores recycling essential nutrients. They are a defining component shaping ecosystems, forestry and agricultural practices, and underpinning food security and the global economy [4,9].

### 1.2. Circadian Rhythms and Their Fundamental Importance

Organisms on Earth have evolved under the influence of a 24 h day/night cycle. As such, biological rhythms play a cardinal role in regulating the biology and ecology of organisms across the entire biosphere. This can be seen in the flowering time in plants, and in feeding rhythms and sleep/wake cycles in animals [10,11]. Circadian clocks are the internal timekeeping systems responsible for generating autonomous ~24 h rhythms. These clocks are entrained by external cues (zeitgebers), including light and temperature, but also exhibit free-running rhythms that persist under constant conditions without time-of-day cues [12].

The circadian clock function involves three distinct components: (i) input pathways, which perceive environmental cues; (ii) the central oscillator, responsible for self-sustaining molecular rhythms featuring canonical clock components; and (iii) output pathways, which regulate downstream biological processes governed by the clock [11,13].

Temporal alignment of clock-controlled rhythms in both insects and plants contributes to the ecological dynamics of herbivory [14,15]. Insect circadian clocks determine daily rhythmicity in physiology and behaviors such as locomotion, feeding, mating, and oviposition, as well as developmental events, including hatching, pupation, and eclosion [16]. Plant circadian clocks regulate key processes, including stem elongation, leaf expansion, flowering, leaf movement, and defense mechanisms against herbivores and environmental stresses [10].

### 1.3. Impacts of Insect Herbivory on the Economy and Global Food Security

Insects have a multifaceted relationship with the agricultural industry. Some sectors of agriculture, such as fruit growing, are reliant on insect pollination, while all are negatively impacted by insect herbivory, oviposition, and by insects as disease vectors. An estimated USD 70 billion are lost yearly to invasive insects [17], and reportedly a staggering 38% of crop losses in agriculture are to insect pests [18], not including the effects of postharvest damage and/or of diseases spread by insect vectors. In response, the use of pesticides to chemically control insect populations has substantially increased over the past decades [19].

Unfortunately, the use of pesticides has resulted in increased resistance in insect populations, as well as collateral harm across natural populations, including insect pollinators and natural enemies [20]. Moreover, exposure to insecticides has been linked to negative impacts on human health [21]. Increased pesticide resistance has incentivized the development of increasingly toxic treatments, so that in recent years each new generation of insecticides has been associated with more drastic off-target effects on other insects [22]. 

In this review, we will set out the aspects of insect and plant circadian biology that relate to herbivory, before exploring the rhythmic interactions within insect–plant systems that govern the temporal dynamics within them. We will not only illustrate the layers of complexity that make up the circadian regulation of herbivory within multitrophic systems but also make the case that dissecting the underpinning molecular mechanisms will help develop insect management strategies, with applications in agriculture and forestry.

## 2. Circadian Clock Mechanisms

### 2.1. Basic Architecture of Circadian Timekeeping Systems

Circadian clocks are systems for daily timekeeping that help to temporally align biological functions in an optimized daily schedule. They are defined by (1) ~24 h periodicity, (2) entrainment to external environmental cycles, (3) self-sustained oscillations under constant conditions, (4) temperature compensation of their period length, and (5) the generation of output via the rhythmic control of biological functions (e.g., [23]). Properties 1, 3, and 4 are contributed by the molecular oscillator of circadian clocks, while input and output pathways provide properties 2 and 5, respectively. Circadian clocks that meet all of these criteria are found across a wide variety of organisms, including prokaryotes such as cyanobacteria, as well as unicellular and multicellular eukaryotes. The recent demonstration of circadian clock function in *Bacillus subtilis* suggests that additional circadian clocks are yet to be discovered [24,25]. There is relatively little overlap in the molecular circadian clock components identified for well-studied systems in cyanobacteria versus plants versus animals with a higher level of conservation within these groups (e.g., between insect and human clocks). Protein phosphorylation cycles are integrated into all well-studied molecular circadian clocks. In most eukaryotes, these cycles are coupled to transcriptional and translational rhythms, as well as regulated proteolysis, whereas, in cyanobacteria, the core phosphorylation oscillator can function independently of transcription and proteolysis, which mainly modulate amplitude and output [23,26].

### 2.2. Insect Clocks

Molecular clock circuits in insects reside in a subset of brain cells, as well as in peripheral tissues elsewhere in the body. Clock-bearing neurons are commonly found in both lateral and dorsal locations of the insect brain [27,28,29,30,31,32,33,34,35]. *Drosophila melanogaster* has most recently been estimated to harbor a complement of 240 neurons divided over lateral (including ventral lateral, dorsal lateral, and lateral posterior subtypes) and dorsal type 1, 2 and 3 neurons with a growing number of subtypes across these categories [36]. The circadian pacemaker function was mapped to the accessory medulla in the lateral brain of cockroaches [37]. The equivalent region in the *D. melanogaster* brain is home to extensive connections between visual pathways and ventral lateral clock neurons [36]. In the adult *D. melanogaster* brain, a set of eight small ventral lateral neurons expressing the neuropeptide PIGMENT DISPERSING FACTOR (PDF) acted as the morning circadian pacemakers in anticipation of dawn [38], while a subset of PDF-negative lateral neurons was found to act as evening pacemakers in anticipation of dusk [39,40,41].

#### 2.2.1. Core Clock Components in Insects

**Figure 1:**
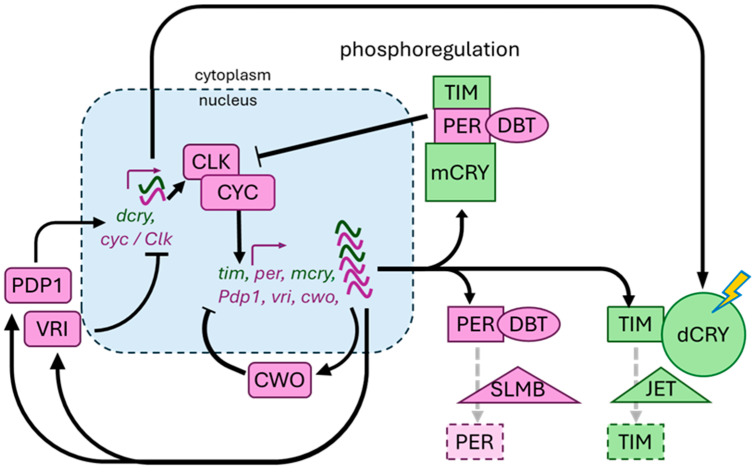
Model for the core molecular circadian clock circuit in insects. Most of the supporting evidence for this model comes from experiments in *Drosophila melanogaster* [71,72], while studies of other insects have confirmed that the complement of clock components is variable across different insect lineages [73,74,75]. Positive regulation is represented by solid black arrows, negative regulation by bar-headed black arrows, protease-mediated turnover by dashed gray arrows. Genes are represented in italic font and accompanied by right-angled arrows. Transcripts are represented by sinusoidal lines. Kinases are represented by ovals, photoreceptors by circles (with lightning bolt), F-box proteins by triangles, and transcriptional regulators are indicated by rectangles, with DNA-binding activity further annotated by rounded corners. Genes and proteins in purple are thought to be (near-)universal across insect clocks, while those in green are absent from some lineages. The heterodimeric bHLH-PAS transcription factor CLK/CYC activates transcription via E-box enhancer elements in a series of target genes that includes the *period* (*per*) gene. PER protein is bound and progressively phosphorylated by the casein kinase I homolog DBT, which impacts PER turnover via the F-box protein SLMB and the proteasome. PER acts together with co-factors including TIM and/or transcriptionally repressive mCRY to negatively feedback on the transcriptional activity of CLK/CYC. This process is modulated by phosphoregulation by additional kinases and phosphatases, which together with DBT help control the stability and nuclear entry of negative feedback regulators. Insects that express TIM, along with *Drosophila*-type CRYPTOCHROME (dCRY) and the F-box protein JET, use these components for light-mediated entrainment of the core oscillator. Blue light induces a conformational change in dCRY, triggering interaction with TIM and JET-mediated light-dependent turnover of TIM by the proteasome. Additional negative feedback is provided via the binding of E-box enhancers by the competitive inhibitor CWO encoded by another CLK/CYC transcriptional target gene. Finally, two additional CLK/CYC-regulated genes encoding bZIP transcription factor, *vri* and *Pdp1*, regulate clock-controlled genes negatively and positively, respectively, via V/P box enhancers.

Insect circadian clocks described to date feature delayed negative feedback loops of gene expression (Figure 1). Since its role in the discovery of clock genes [42], the fruit fly *D. melanogaster* has become instrumental as a model for the molecular timekeeping circuits of the circadian clock. Much of our current understanding of the genetic and molecular underpinnings of daily timekeeping has come from the research regarding this genetic model. Nevertheless, it has become clear that the clock of *Drosophila* and other flies in the suborder *Brachycera* is highly evolved and has some features distinguishing it from that of most other insects [43]. We will, therefore, focus on aspects of the molecular clock circuits that appear to be shared widely across insects and then comment on where insect clocks diverge. Central to insect clocks is the role of the clock/cycle (CLK/CYC), a heterodimeric basic helix–loop–helix Per-Arnt-Sim domain (bHLH-PAS) transcription factor, as a key activator of circadian oscillation of transcription [44,45,46]. The transcriptional program initiated by the CLK/CYC complex triggers at least three feedback loops that help establish oscillations in its activity: (1) inhibition of CLK/CYC transcriptional activity by complexes that include period (PER) proteins along with the doubletime (DBT) kinase, as well as obligatory co-factors [47,48,49]; (2) competitive inhibition of CLK/CYC binding at E-box enhancer sites by the bHLH orange domain transcription factor clockwork orange (CWO) [50,51,52]: (3) the generation of anti-phase transcriptional oscillations in genes encoding CLK and/or CYC [53,54]. In the first of these feedback loops, PER/DBT complexes need to interact with timeless (TIM) and/or transcriptionally repressive mammalian-type cryptochrome (mCRY) [55,56,57,58,59]. Phosphoregulation by DBT and other kinases and phosphatases plays a role in both the accumulation and nuclear entry of negative feedback complexes [60,61,62,63,64,65]. In this context, the phosphorylation-induced turnover of PER by the proteasome is particularly relevant. This process involves the recognition of specific phospho-PER isoforms by the conserved F-box protein SUPERNUMERARY LIMBS (SLMB) [66,67]. The second feedback loop enhancing oscillator amplitude features CWO, which both competes with CLK/CYC at its E-box binding sites to decrease transcriptional activation [50,51,52] and induces higher peak CLK/CYC activity levels by repressing the expression of the CLK/CYC inhibitor CLOCK INTERACTING PROTEIN CIRCADIAN (CIPC) [68]. Finally, molecular circadian rhythmicity is further reinforced by the rhythmic regulation of the *cyc* and/or *Clk* genes. For example, the *Drosophila Clk* gene receives negative and positive feedback via the basic leucine zipper transcription factors VRILLE (VRI) and PAR-DOMAIN PROTEIN 1 (PDP1) [53,54]. It should be noted, however, that the arrhythmic behavioral mutant phenotypes for *vri* and *Pdp1* in *Drosophila* were found to depend on defects in clock output rather than their regulation of the *Clk* gene [69,70]. Nevertheless, the rhythmic accumulation of *Clk* and/or *cyc* transcripts in antiphase to those of their target genes may further promote oscillations between CLK/CYC active and inactive states.

In most insects, the molecular clock features both TIM and mCRY, with the former lost in Hymenoptera and termites and the latter lost in higher Diptera [76]. In addition, in most insects—with the exception of a group of higher Diptera—*cyc* rather than *Clk* is thought to carry the transcriptional activation function for the CLK/CYC complex and exhibit the more relevant transcriptional cycling (reviewed in [43]).

#### 2.2.2. Insect Clock Entrainment and Synchronization

Insect circadian clocks have been demonstrated to synchronize to environmental rhythms in light, temperature, restricted feeding, and social cues, as well as mechanical and chemical stimuli. Generally, light has been studied most extensively and has been found to be a key environmental time cue or zeitgeber for most adult insects. Light input into the circadian clock can be cell-autonomous via the blue light photoreceptive dCRY or non-cell-autonomous, e.g., via input from visual organs. In *D. melanogaster*, both light input pathways are important, with dCRY directly responsible for blue light-mediated entrainment of both peripheral clocks and brain clocks and the visual organs contributing to the entrainment of brain clocks and associated circadian outputs across a wider set of wave lengths [77,78,79,80,81,82,83]. Upon activation by blue light, dCRY binds TIM and promotes its recognition by the F-box protein JETLAG (JET), which leads to ubiquitination and proteasomal turnover of TIM [78,84,85,86] (Figure 1). This in turn reduces PER-mediated negative feedback of CLK/CYC and accounts for the light-mediated phase resetting of the molecular oscillator [56,57]. The genomic loss of *dcry* has been observed for some insect groups, including Hymenoptera, crown Coleoptera, Phthiraptera, Pentatomorpha, Cimicomorpha, cockroaches, and termites [76]. It is tempting to speculate that there is a diminished dependence on light as the dominant zeitgeber for insects in these taxa in association with evolution in relatively ecological dark niches (nests, hives, bark, leaf litter, hosts) or acquisition of more prominent alternative thermal, social chemical, or mechanical entrainment mechanisms [43,76,87,88,89,90,91]. A recent analysis noted that changes in *tim*’s functional features co-occur with the gene loss of *dcry* or *jet*, further emphasizing the joint role of these components in regulating light-mediated circadian entrainment [92].

Temperature entrainment has been identified across a range of insects [88,91,93,94,95,96,97,98,99]. The diel transcriptome of *Drosophila melanogaster* features not only prominent temperature-driven regulation but also substantial temperature-entrained circadian responses [100]. Temperature cycles differentially impact *Drosophila melanogaster* core clock transcripts and proteins [100,101,102,103], and peripheral clocks in isolated tissues readily synchronize to temperature cycles [104]. Nevertheless, the *Drosophila* brain clock employs dedicated sensory input pathways featuring the chordotonal organs [105], ionotropic receptors (Ir25a) [106], and TRP channels (e.g., PYREXIA [107,108]). Notably, the *Drosophila* chordotonal organs not only mediate entrainment to temperature but also to mechanosensory cues generated by vibration [109]. Thus, the temperature-driven modulation of locomotor activity could contribute to entrainment of the circadian clock via mechanosensory signaling from the chordotonal organs in the legs. The principle that TRP channels play a role in temperature input for circadian rhythms is widely supported, e.g., cold-sensing TRPM8 in mouse brown adipose tissue [110]. However, the extent to which molecular temperature entrainment pathways in other insects match those identified in *Drosophila* largely remains to be determined.

Insect circadian clocks have also been demonstrated to respond to social cues (e.g., [99,111,112]), with prominent examples of social entrainment identified in eusocial insects [87,113,114,115,116]. Olfactory cues impact social synchronization in *Drosophila* [87], while non-contact zeitgebers (e.g., via volatiles, vibrations) have been found for social circadian entrainment in honeybee hives [87].

#### 2.2.3. Clock Output Pathways in Insects

One of the most obvious ways for circadian clocks to generate output is via the control of circadian gene expression rhythms across large segments of the genome by core clock transcription factors. This was experimentally confirmed by the determination of the circadian transcriptome of *Drosophila melanogaster* [117,118,119,120,121]. Although *Drosophila* core clock genes oscillate throughout clock-bearing tissues, most clock-controlled genes were found to exhibit tissue-specific rhythmicity [122,123]. The interaction of core clock transcription factors with local transcriptional regulation is thought to be instrumental in the generation of tissue-specific circadian transcript oscillations [122,123]. While output can, thus, be generated by clocks in a cell-autonomous manner throughout the body, intercellular signaling is also employed to regulate both synchrony and output. In particular, neuropeptide signaling in the brain has been studied extensively in this respect [124]. The most prominent example of an impactful circadian neuropeptide is PDF, which is required for sustained synchronous rhythmicity across the brain clock neurons in the absence of environmental entrainment, as well as for correctly timed behavioral anticipation of dawn and dusk [38,125,126]. Of note, insect brains with a stronger coupling of circadian oscillators across brain hemispheres feature PDFergic commissural connections [127]. Studies across different insect species collectively indicate that PDF couples photoperiodic and thermal seasonal timing cues to insect diapause by signaling to neurosecretory cells in the pars intercerebralis (PI) [128,129,130,131,132,133,134,135,136].

Although the molecular circadian phase is relatively synchronous across the *Drosophila* brain clock circuit, subsets of clock neurons exhibit specific peak phases in their activity, as indicated by calcium sensors [137]. Along with light input, PDF from dawn-active circadian pacemakers delays the activity of the evening circadian pacemakers until dusk, while another neuropeptide, SHORT NEUROPEPTIDE F (sNPF) from both morning and evening circadian pacemaker cells controls the late-night activity peak in dorsal neurons of type 1 (DN1) [138]. Clock-controlled calcium rhythms were also traced to downstream cell types, including dopaminergic and neuroendocrine clusters linked to the control of locomotor activity, mating, sleep, and feeding [139,140]. These observations reinforce the findings from parallel studies identifying peptidergic output pathways connecting DN1 and dorsolateral LNd clock neurons directly to subsets of neurosecretory cells in the PI [141] and PDFergic morning pacemaker small ventral lateral neurons (sLNvs) indirectly to a pair of LEUCOKININ (LK) peptidergic cells in the lateral horns [142]. PDF also connects to ALLOSTATIN A (ASTA)-expressing peptidergic neurons in the posterior lateral protocerebrum that promote sleep and reduce feeding [143]. The downstream PI clusters secrete *Drosophila* insulin-like peptides (DILPs), DIURETIC HORMONE 44 (DH44), or SIF AMIDE (SIFA); of these, DH44 has been implicated in the control of circadian locomotor behavior via a further peptidergic relay involving HUGIN [144], while DILPs and SIFA regulate sleep and feeding in a complementary manner with the latter also implicated in the control of mating [145,146,147,148]. On the other hand, LK from the LKLH cells in the lateral horns mediates rhythmic locomotor activity and metabolic modulation of sleep [142,149]. It will be of interest to complement these discoveries in *Drosophila* with analyses of homologous/analogous circadian output pathways across other insect species.

### 2.3. Plant Clocks

Plants employ their circadian clocks to comprehensively optimize their development, growth, and metabolism [150,151]. Large parts of the plant genome exhibit circadian transcriptional regulation [152,153], which mediates widespread circadian control of plant physiology. The hierarchical organization of the plant clock is not as clear cut as in animals. Clocks in different plant tissues exhibit varying degrees of coupling to one another, with variation in robustness and timing between each tissue. Local and distal communication between tissues contributes to the optimal timing of physiology and gene expression within the clock, supported by signaling within the vasculature [154]. Hormones, sugars, and mRNAs of output pathways such as PSEUDO RESPONSE REGULATORS (PRRs) serve as messengers via the phloem [155,156,157]. There is no singular coordinator of the total plant phase, and thus no distinguishable central or peripheral clocks. Manipulation of some clocks can influence the phasing of other clocks, underscoring the concept of coupling, for example, in *Arabidopsis,* the shoot apical meristem clock influences the phase and robustness of clocks in the roots [155], possibly communicated by the long-distance transport of ELF4 [157].

#### 2.3.1. Core Clock Components in Plants

**Figure 2:**
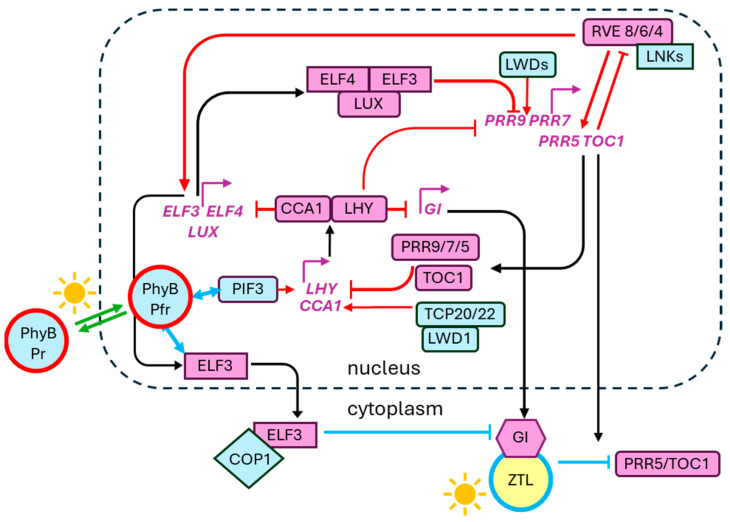
Molecular organization of the plant clock in *A. thaliana*. Circles denote photoreceptors, with the outline color indicating colors of light they are sensitive to; rectangles are transcriptional regulators, with rounded corners additionally indicating DNA binding capacity; the diamond shape represents an E3 ubiquitin ligase; and a hexagon denotes a protein with no known functional domains [167]. Red arrows indicate transcriptional regulation, green indicates a conformational change and nuclear translocation, and blue indicates various protein interactions. Lastly, purple shading indicates cycling proteins, blue indicates non-cycling, and yellow indicates constitutive expression but rhythmic stabilization, ultimately leading to a cycling abundance in the cytoplasm [168].

*Arabidopsis thaliana* is the most extensively studied model for understanding the plant circadian system [158,159,160]. Its clock architecture, like that of insects, is composed of interlocking transcription–translation feedback loops (TTFLs) that ensure a precise and robust rhythm [161,162,163] (Figure 2). These multiple interlocking loops maintain robust rhythmic gene expression under both diel (entrained) and free-running (constant) conditions [164]. While mathematical models have shown that basic circadian timing can emerge from simpler systems, experimental work reveals that these secondary loops become critical for preserving amplitude and rhythmicity under stress conditions, such as suboptimal temperatures [165,166].

At the center of this system is the expression of two MYB-like transcription factors at dawn, CIRCADIAN CLOCK ASSOCIATED 1 (CCA1) and LATE ELONGATED HYPOCOTYL (LHY) [161,169], in part driven by the positive regulation of CCA1 by LIGHT-REGULATED WD1 (LWD1) and TEOSINTE BRANCHED 1-CYCLOIDEA-PCF20 (TCP20) and TCP22 transcriptional co-activation [170]. Once expressed, CCA1 and LHY heterodimerize, forming a morning complex that imposes transcriptional repression by binding the evening element on the promoters for genes encoding PSEUDO-RESPONSE REGULATORS (PRRs) PRR9, PRR7, PRR5, and PRR1 or TIMING OF CAB 1 (TOC1), as well as GIGANTEA (GI) and the evening complex [161,162,169,171], which includes EARLY FLOWERING 3 (ELF3), ELF4, and LUX ARRHYTHMO (LUX; also known as PHYTOCLOCK1 or PCL1) [161,162,169,171,172]. PRR9, 7, 5, and TOC1 are expressed sequentially throughout the day. To achieve this, NIGHT LIGHT–INDUCIBLE AND CLOCK-REGULATED1 (LNK1) and LNK2 interact with MYB-like REVEILLE (RVE) transcription factors and are recruited to the promoters of PRR proteins to promote their transcription [173]. Each of the PRRs exerts a repressive effect on CCA1 and LHY transcription, so that their activity is limited to the morning [161,169,174].

GI protein accumulates slowly during the day in the cytoplasm and stabilizes ZEITLUPE in a blue-light-dependent manner [168]. Briefly, the sunlight around midday contains a significant proportion of blue light that activates ZTL during the day, whereas the evening sunlight is enriched with longer wavelengths and red light that activate the red-light receptor, phytochrome B (PhyB). Towards dusk, once the daylight shifts red, ZTL and GI disassociate, and ZTL promotes the degradation of PRR5 and TOC1 complexes [175]. Also, towards the late evening, the components of the evening complex are expressed, driven by the same LNK-RVE system as the PRRs [173]. Throughout the night, the evening complex binds to the promoters of PRR9 and PRR7 via the LUX binding site (LBS) to repress their transcription [172], thereby relieving CCA1 and LHY of transcriptional repression by the PRR9/7/5 complex and TOC1 so that CCA1 and LHY complexes accumulate in the cell in the morning and the cycle can begin again at dawn.

Importantly, while *Arabidopsis thaliana* remains the foundational model for understanding plant circadian networks, translating these insights to agriculturally relevant species is essential for addressing crop-specific traits and adaptive responses.

#### 2.3.2. Plant Clock Entrainment and Synchronization

The plant clock entrains to a number of zeitgebers, including light and temperature, but primarily it relies on light for resetting the clock and determining time of day and season [176]. The light input pathways to the plant clock are managed via phytochromes, cryptochromes, and blue-light photoreceptor ZTL. By using photoreceptors that respond to different wavelengths of light, the plant clock can be finely tuned to the exact time of day. PhyB is the main photoreceptor for red light input to the clock, and among its functions is the interaction with PHYTOCHROME INTERACTING FACTOR 3 (PIF3) to positively regulate CCA1 expression at dawn [177] (Figure 2). Alongside this, it interacts with ELF3 around dusk to promote the degradation of GI once it is no longer bound to ZTL [169,176,178]. The ZTL function, described above, is negatively regulated by the presence of blue light, and as such it plays a key role in the phasing of the clock by delaying the degradation of TOC1/PRR5 and subsequent cascade triggering CCA1/LHY expression until the appropriate time of day [168]. For a more comprehensive and detailed review of light input pathways to the plant clock, see Sanchez, Rugnone, and Kay [176]. 

The role of temperature in plant clock entrainment has yet to be fully understood. Early work identified that *PRR7* and *PRR9* have a somewhat overlapping function in the role of temperature entrainment [179], and more recent mathematical modeling has supported this finding, alongside reportedly concluding that the role for temperature entrainment is in conjunction with light entrainment rather than as a solo zeitgeber [180]. Investigation of a *heat shock protein 90* (*hsp90*) mutant that exhibits an exaggerated period lengthening following temperature cycle entrainment revealed HSP90 interaction with one or more components from the morning loop, CCA1, LHY, and PRR7, as a candidate for a temperature entrainment mechanism [181]. Ultimately, the exact process by which the clock is entrained by both light and temperature in combination has yet to be revealed.

#### 2.3.3. Plant Clock Outputs

Plant clock outputs are coordinated primarily by gene expression: morning and evening phase complexes regulate the transcription of a respective set of genes involved in hormone biosynthesis, photosynthesis, and sugar metabolism [13]. The phasing of these clock genes determines the timing of the transcriptional regulation of non-clock genes by the transcription factors that make up the clock [150,152,153]. Core clock transcription factors have suites of identified targets that they can act on via direct binding, co-factors, or through epigenetic regulation [182,183,184]. Specific pathways and targets for transcriptional regulation have been identified for several mechanisms and pathways that exhibit circadian rhythmicity. In the following sections, we will describe what is known about circadian regulation in the areas of plant function relevant to herbivory, including plant metabolism, the synthesis and signaling of essential phytohormones, and the regulation of plant defenses with a focus on direct and indirect chemical defenses.

## 3. Plant Circadian Biology Relevant to Herbivory

### 3.1. Daily Rhythms in Plant Metabolism

Chloroplasts and mitochondria sit at the core of plant metabolism, characterized by the interplay between photosynthesis and respiration. The function of both mitochondria and chloroplasts is tightly coupled to the circadian clock [13,185].

One of the most vital functions of mitochondria is the production of cellular energy, via cellular respiration by the tricarboxylic acid (TCA) cycle and generation of ATP via the electron transport chain (ETC), and both are clock regulated in *Arabidopsis* [185]. Several core clock proteins have been implicated in the function of the TCA cycle, including CCA1, TOC1, and the PRR family [186,187]. Misexpression of all three resulted in changes to the accumulation of TCA intermediates, most commonly succinate, fumarate, and malate [186]. Most notably, TOC1 was found binding to the fumarase 2 promoter [187], offering a candidate for a direct mechanism of TCA cycle regulation by the plant clock. *TOC1* misexpression also unveiled possible clock regulation of the ETC, with a *TOC1*-overexpressing line exhibiting significant changes to the otherwise circadian expression of genes encoding proteins that make up part of the ETC [187]. Both *TOC1*-overexpressing and *toc1-1* mutant lines exhibited ATP/ADP ratios that deviated significantly from the wildtype [187], suggesting the circadian regulation of cellular energy in *Arabidopsis*; however, the direct mechanisms by which this could occur have not yet been uncovered.

The circadian regulation of photosynthesis contributes to the optimal functioning of a plant. Plants fix CO_2_ either primarily during the day or during the night, a process which is known as crassulacean acid metabolism (CAM) [188]. In both scenarios, a tight circadian regulation of the mechanisms behind CO_2_ fixing is required.

Carbon fixation carried out by ribulose 1,5-bisphosphate carboxylase/oxygenase (RuBisCO) is thought to be an ideal candidate for circadian regulation [189]. RuBisCO acts within the Calvin cycle, and its activity is restricted to daytime via nighttime inhibition by 2-carboxy-D-arabinitol 1-phosphate (CA1P) and ribulose-1,5-bisphosphate (RuBP) [189,190]. The circadian regulation of RuBisCO activity has not yet been fully explained, although several mechanisms have been suggested. Transcripts of the gene encoding RuBisCO activase (RCA) and the small subunit of RuBisCO were both reported to oscillate [191]. In kidney bean (*Phaseolus vulgaris*), RuBP concentrations reportedly exhibited diurnal rhythmicity [192], which may translate into the rhythmic inhibition of RuBisCO activity. More recently, PRR9, 7, and 5 were described to negatively regulate the chlorophyll biosynthesis pathway among others [186], which could be a mechanism of circadian regulation in light fixing, upstream of the RuBisCO function.

Circadian rhythms in gas exchange in obligate CAM plants were first reported by Ritz and Kluge [193], who noticed rhythms in chandelier plant (*Kalanchoë tubiflora*) net CO_2_ uptake, leaf conductance, and transpiration that were sustained for ~7 days in constant light. In addition to this, they also observed that PEP-carboxylase (PPC) sensitivity to feedback inhibition by malate showed circadian rhythmicity [193]. This process is one of the key mechanisms by which CAM plants can support nighttime carbon fixation, phosphorylation of PPC by PPC-kinase (PPCK), reducing their affinity for allosteric inhibition by malate [194]. Since then, it has been identified that the changes in malate sensitivity are down to circadian rhythms in PPCK abundance, the transcript of which is known to oscillate rhythmically in lavender scallops (*Kalanchoë fedtschenkoi*), a rhythm that is maintained in constant light [194]. Moreover, the silencing of *PPCK1* was reported to dampen the free-running gas exchange oscillations in *K. fedtschenkoi* in only 2 or 3 days, drastically faster than wildtype plants, which maintained gas exchange rhythms for more than a week [194].

Unsurprisingly, primary metabolism also appears to be clock-regulated, either directly or indirectly. RVE4, 6, and 8 have been implicated in organic acid and lipid synthesis [195] and CCA1/LHY reportedly bind to the *β-ketoacyl-[acyl carrier protein] synthase III (KASIII*) promoter, which encodes a synthase enzyme that catalyzes the early stages of fatty acid production in *Arabidopsis* [196]. Diurnal oscillations in the accumulation of both starch and sugars have also been widely reported [187,195,197,198]. Starch and glucose synthesis gene transcripts accumulate rhythmically in upland cotton plants [198]. These rhythms appear to be conserved across species; a study comparing lettuce cultivars found that sucrose synthase and both acidic and neutral invertase genes all exhibited diurnal rhythms that remained robust in constant light [197]. Core clock components have also been implicated directly. Multi-omic analysis of the *rve 4 6 8 A. thaliana* line, mutant for clock transcription factor family *REVEILLE*, revealed a potential involvement in the regulation of starch content at ZT0 (lights on) [195]. A knockdown of *GI* in Chinese cabbage (*Brassica rapa*) also uncovered aberrations in total starch and sugar content [199], while *cca1* lines had a lower concentration of fructose, and *d974* (mutant for *PRR9*, *7*, and *5*) had a higher concentration of sucrose [186]. It is important to note that the impact of mutation or knockdown on these clock genes does not necessarily indicate a direct link between them and part of the sugar or starch synthesis pathways; instead, it implicates circadian timekeeping, since the impact of knockdown or mutation on one core clock element can affect the phasing and activity of all others, and there are likely other intermediate clock controlled pathways upstream of sugar and starch synthesis.

It follows that secondary metabolites, downstream of other metabolic processes, also often exhibit circadian rhythmicity in plants. The most relevant to herbivory are the secondary metabolites involved in plant defense. These include nicotine, glucosinolates (GSLs) that act as a key part of the “mustard oil bomb” mechanism in crucifers, and herbivore-induced or oviposition-induced plant volatiles (HIPVs and OIPVs, respectively), which are signaling molecules released in response to insect herbivore feeding or oviposition [200].

### 3.2. Temporal Regulation of Plant Defense Compounds

#### 3.2.1. Plant Defense Strategies

Plant defense strategies to deter herbivory come in physical and morphological changes to plant architecture such as trichomes and thorns, as well as in the accumulation of secondary metabolites, including chemical toxins to deter animals [200,201]. The latter can be faster and is known to be regulated both directly and indirectly by plant defense hormones. Upon damage to the plant, phytohormones jasmonic acid (JA), ethylene, and salicylic acid (SA) are upregulated, triggering signaling cascades that mediate timing, magnitude, and composition of a cocktail of defensive compounds [202]. JA and ethylene induce secondary metabolites and proteinases that are effective in deterring herbivores, whereas SA induces the microbial defense pathway. At the onset of herbivory, fragments of damaged plant tissue and herbivore elicitors (e.g., olfactory secretions) are recognized as herbivore-associated molecular patterns (HAMPs), which trigger the induction of defense-associated hormones [200,202,203]. This is usually followed by an induction of SA-regulated microbial defense since herbivores carry microbes and viruses [204]. Complex interplay between JA, ethylene and SA induction is thought to determine which downstream pathways are activated, which may allow the plant to tailor defense to one or multiple specific insect infestations [205,206], and crosstalk between these phytohormones and the circadian clock contributes to the temporal regulation of defensive responses [207,208].

The plant clock ensures plants invest their energy into biomass production and/or defense at the most effective time of day [209,210]. The defense comes at a cost to growth and reproduction; therefore, anticipating vulnerability supports plant fitness by only requiring defense metabolite production when necessary [211]. Since biotic attacks on plants are often preferentially diurnal or nocturnal, i.e., rhythmic [15,211,212,213], it is not surprising that plant defense metabolite accumulation [15,212,214,215] and HIPV release during infestation [14,216,217,218,219,220] have been found to be rhythmic and regulated—at least in part—by the circadian clock.

#### 3.2.2. How Does the Clock Regulate Defense?

**Figure 3:**
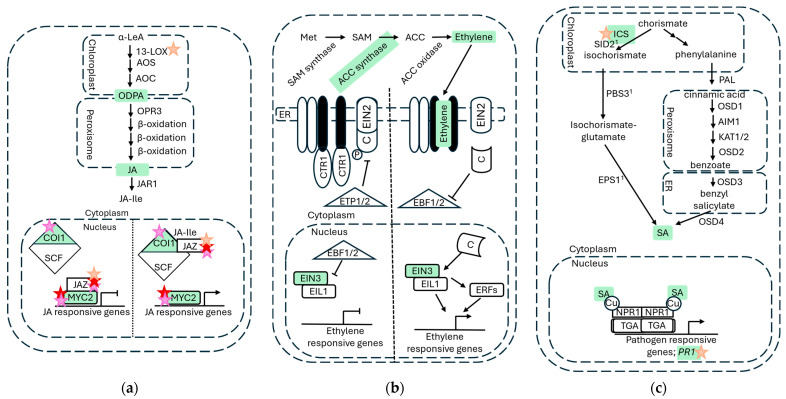
Circadian regulation of defensive phytohormone biosynthesis and signal transduction. (**a**) Jasmonic acid, (**b**) ethylene, (**c**) salicylic acid. Positive regulation is represented by solid black arrows, negative regulation by bar-headed black arrows. Genes are accompanied by right-angled arrows. E3 ubiquitin ligase complexes are indicated by diamonds, F-box proteins by triangles, kinases by ovals, and transcription factors are indicated by rectangles with rounded edges, indicating DNA binding activity. The unmarked white and black ER transmembrane proteins in (**b**), represent RTE1 and ETR1, respectively. Green background indicates compounds described to accumulate rhythmically, pink stars indicate clock protein binding sites, red stars indicate circadian regulation of a compound, orange stars indicate compounds identified to be differently abundant in a clock mutant, and ^1^ denotes enzymes exclusive to Brassicaceae.

There are three mechanisms by which the clock may regulate plant defenses: (1) regulation of JA, ethylene, and SA biosynthesis; (2) gating of phytohormone signaling; (3) direct regulation of the defense compounds themselves (Figure 3). While there is clear evidence of (1) and (2), there are two schools of thought on (3). It is possible that all rhythms seen in defensive metabolites are simply a result of clock gating of signal transduction via JA/ethylene/SA plant hormones. Alternatively, the binding of clock components directly to the genes encoding the enzymes for defense compound biosynthesis has been suggested, e.g., *CYP79* genes in both *A. thaliana* and tea plants (*Camellia sinensis*) [220,221].

Rhythms in accumulation have been observed in JA, ethylene, and SA (Figure 3) [15,208,222], but it is not yet clear for all exactly how this is controlled. The JA precursor ODPA was found to exhibit rhythmic accumulation in wild tobacco (*Nicotiana attenuata*) [212], and, upstream of this, the genes encoding two 13-LIPOXYGENASEs, *LOX3*, and *LOX4* were identified as significantly upregulated in a *lux* mutant line of *A. thaliana* [223], implicating regulation by the evening complex via LUX as a possible mechanism of JA biosynthesis regulation. Similarly, ethylene accumulation rhythms exhibited a close correlation with light- and clock-modulated transcript rhythms of *ACC synthase 8* (*ACS8*) [222]. In line with this, transcripts of ACC-induced and -repressed genes were found to accumulate rhythmically and antiphase to one another, peaking during the late day and late night, respectively [153]. SA biosynthesis may be clock regulated via the major synthesis enzyme ICS, which controls the first step of the isochorismate pathway, the predominant pathway in *Arabidopsis* [224], as it was downregulated in a *lux* mutant line of *A. thaliana* [223]. Interestingly, CCA1 has also been identified as a candidate for *ICS* expression regulation. The hybrid offspring of the Col-0 and Sei-0 ecotypes exhibited an 8 h phase advance in the expression of *ICS*, a phenotype which was lost when this hybrid was generated from *cca1-1* mutant lines [183]. Moreover, there was also significantly lower *ICS* expression in these *cca1-1* hybrids [183]. These findings implicate the clock in not only absolute levels of *ICS* expression but also in its phasing. While CCA1 and LUX have both been suggested as candidates for the transcriptional regulation of *ICS* expression, it is not yet clear if either of them are directly responsible. Since each component of the core clock feeds back directly or indirectly into all others, these results simply suggest that some stage of the clock is involved in regulating *ICS* expression and may be the process by which SA is rhythmically generated.

While rhythms in JA, ethylene, and SA accumulation are relevant to active herbivory, the temporal regulation of defense mechanisms is also mediated by clock gating of the signal transduction pathways, which is best studied in the JA signaling cascade. Clock regulation of essential components required for a specific signaling mechanism means that the magnitude of a defensive response, or the sensitivity of a plant defense to wounding signals, is different depending on the time of day. One of the master regulators of JA signaling, MYC2 transcription factor protein, is stabilized and accumulates from midday until dusk [225]. *MYC2* transcription peaks at dusk and both the protein and transcript accumulation are regulated by the circadian clock via Time for Coffee (TIC) protein, in which TIC negatively regulates MYC2 by promoting the protein degradation. Briefly, TIC is a nuclear protein and the clock regulator found interacting with CCA1/LHY to form a morning complex [226]. The *MYC2* promoter in both *A. thaliana* and *C. sinensis* contains LUX binding sites (LBS), and in the former it is bound at the second of its two LBS by LUX and ELF4, two components of the evening complex in the plant clock [220,227]. MYC2 could be negatively regulated by TIC in the morning and positively regulated by components of the evening complex in the evening; however, this rhythm does not align with the typical sensitivity of jasmonate signal transduction, thought to peak during the day rather than overnight. MYC2 is also negatively regulated by JASMONATE ZIM DOMAIN (JAZ) proteins, to mitigate activation of downstream defense pathways at inappropriate times [228]. *JAZ1* and *JAZ5*, like *MYC2*, were identified to have LBS in their promoters in *A. thaliana*, and it was later reported that LUX could bind to the promoter of *JAZ5* [223,227]. In bushy tobacco (*Nicotiana benthamiana*), the clock protein ZTL was reported to interact independently with the JAZ protein, contributing to the synthesis of nicotine [229]. The role of ZTL in plant defense was corroborated by the presence of ZTL in *N. attenuate*, conferring resistance against the generalist herbivore African cotton leafworm (*Spodoptera littoralis*). While ZTL transcripts are constitutive, ZTL proteins must be stabilized by GI, and are degraded after it translocates to the nucleus [168]. Therefore, ZTL-JAZ interactions are clock dependent and so is the resulting regulation of nicotine production. Via the regulation of JAZ proteins and MYC2, the clock is able to gate all pathways downstream of jasmonate signal transduction, facilitating one mechanism of temporal control of plant defenses during herbivory. 

It has also been suggested that, for various defense compounds, there could be a direct clock regulation of their respective synthesis pathways. Accumulations of glucosinolates (GSLs), a class of secondary metabolites involved in the “mustard oil bomb” defense exclusive to crucifers [230,231], rhythmically oscillate daily in *A. thaliana* and *Brassicas* [214,221,232]. The mustard oil bomb is characterized by the breakdown of GSLs by plant myrosinase enzymes into toxic compounds [230,231]. GSLs contain a glucose sugar moiety, a β-D-glucopyranose (glucose) residue linked to a sulfate group, and a side chain derived from either an indole, aromatic, or aliphatic amino acid, and each accumulates in distinct but varied circadian patterns in *Brassica* cultivars [232]. 

GSL biosynthesis is regulated by R2R3-MYB transcription factor MYB34, which binds to the promoters of *CYP79B2* and *CYP79B3*. The products of these two cytochrome P450 monooxygenase genes catalyze the first committed step in indole GSL biosynthesis [221,233]. Lei et al. (2019) [221] reported the identification of CCA1-binding sites on the promoters of *MYB34*, *CYP79B2*, and *CYP79B3* genes, although binding has not yet been confirmed. CCA1 overexpression Arabidopsis plants were shown to accumulate approximately 2-fold more indole glucosinolates compared with wildtype [221]. It is clear that the clock, either directly or indirectly, regulates glucosinolate accumulation in *Brassica* crops and *A. thaliana*, but what has yet to be confirmed is whether this occurs through the direct interaction of CCA1 with genes required for GSL biosynthesis or whether this regulation is mediated by the clock gating of JA signaling, which is known to lie upstream of the GSL synthesis pathway [234,235]. One argument could be that the regulation of GSLs occurs both directly and indirectly, since the clock gating of jasmonates is well studied, but GSLs accumulate with circadian rhythmicity even in uninfested/undamaged plants [214,232], at a time that jasmonates are not necessarily signaling. 

HIPVs constitute a means of communicating herbivory status to both natural enemies of herbivores and other plants [202]. Rhythms in HIPV emission have been described in legumes, including *P. vulgaris* and lima bean (*Phaseolus lunatus*), as well as in black poplar, maize (*Zea mays*), and *C. sinensis* [14,216,217,218,236]. These rhythms may represent gating and/or direct regulation by the plant clock [202]. Diurnal rhythms in terpene emissions, for example, exhibit a distinctive “burst” at the onset of the light phase in various plants [216,236], which it has been stipulated could be associated with a sharp increase in JA signaling in *Z. mays* [236]. Notably, it was previously argued that this may also be a result of a light dependent step in *P. lunatus*, especially since JA upregulation occurred during the night as well as the day in damaged leaves [216]. Benzyl nitrile emissions from *C. sinensis* were reported as clock regulated [220], following findings that silencing *CsLUX* (aka *CsPCL1*) resulted in the significant downregulation of *CsCYP79*—required for benzyl nitrile synthesis—and *CsMYC2*. The authors stipulated that, since the promoters of both genes contained LBS, CsLUX could induce the transcription of either one. As with the regulation of GSLs in *A. thaliana*, there is clearly a capacity for direct transcriptional regulation by the clock, but since no interaction between CsLUX or the *CsCYP79* promoter was demonstrated, it is unclear whether this is the mechanism by which benzyl nitrile is regulated. Since HIPV emissions tend only to occur at times of defensive phytohormone signaling [202], it may be that the rhythms observed in the *C. sinensis* benzyl nitrile emission are a downstream effect of the clock gating of JA signaling. 

#### 3.2.3. Functional Effects of Clock Gating on Herbivory Defense

In recent years, more studies have been conducted on the effects of clock gating on herbivory defense. This was previously demonstrated in plant immunity against pathogens, in which the inoculation of *A. thaliana* plants with *Botrytis cinerea* bacteria at different timepoints led not only to different sized lesions but also altered speed of the transcriptional response itself [237]. Applying this to herbivory, the expectation would be that wounding at varying times of day would elicit a defensive response to different degrees, but this has not yet been conclusively demonstrated. While it is useful to understand how gating might work, it is important to translate these results into real-world situations by observing how they shape the interaction between plants and insect herbivores.

In *P. lunatus*, mechanical wounding at night induced a significantly higher jasmonate concentration than during the day [216], but these results were not applied to a specific plant–insect interaction. A wounding entrainment conducted on three *Brassica* crops was used to determine the effects of gating on defense metabolite accumulation, which was then compared with feeding by the generalist cabbage moth (*Mamestra brassicae*) [215]. It was reported that the time of day of wounding resulted in a different composition of GSLs, and that *M. brassicae* feeding was reduced on plants wounded at a specific timepoint. While these results do underscore the point that GSL accumulation is rhythmic, and that the specific blend of GSLs is also dependent on time of day, it was important to determine whether this reflected clock gating of wounding responses rather than innate wounding–independent GSL oscillations. Although *M. brassicae* feeding began and ended at different times of day, it was measured in each case over 24 h, meaning an entire circadian cycle of both the insect feeding rhythms and the plant defense rhythms. *Brassica oleracea* wounded at ZT0 experienced significantly less loss to the leaf area after 24 h of *M. brassicae* feeding [215], which could suggest that the defensive response is induced either to a greater extent or in a more effective way when this crop is wounded at the onset of the light phase. This may be a direct result of clock gating via the jasmonate signal transduction pathway. Since the plant defenses were “primed” before feeding by multiple days of mechanical wounding, this study is not fully reflective of the natural environment, but it opens the door to future investigation into how the timing of plant stress can, via gating, impact a plants’ ability to tolerate or defend against herbivory and mediate insect behaviors.

## 4. Insect Circadian Biology Related to Herbivory

### 4.1. Environmental Time Cues and Seasonality

Insects are the most diverse and widely distributed animal group, inhabiting environments from tropical lowlands to alpine areas, the equator, and polar regions [238]. This ecological breadth suggests that circadian clocks have played a critical role in their evolutionary success by enabling synchronization with varied cyclic environmental changes [11]. The successful colonization of extreme latitudes is likely as a result of their highly plastic clocks, facilitating adaptation to radically different changes in day length between different regions. In temperate climates, synchronization with seasonal changes is especially critical; insects must anticipate adverse conditions like winter and coordinate development or reproduction accordingly [239,240].

Metabolic processes can be downregulated or shut down in response to environmental conditions or for certain life stages; these changes can also occur rhythmically. The transition between life stages has been shown to be regulated by the circadian clock [241,242,243], including for hatching and eclosion [244,245,246]. Preparation for pupation and likely digestive shutdown in silk moths includes gut purging, which occurs rhythmically [247]. Diapause and migratory responses to the environment are also closely linked to circadian clock regulation. A functional circadian clock is crucial for photoperiod-induced diapause [128,248,249]. Shifts in photoperiods can also lead to diapause eggs to be laid by silk moth (*Bombyx mori*), dependent on *per* expression [133], with *tim* and *cry* in *Drosophila triauraria* controlling general diapause [250]. These and other core molecular clock genes have also been shown through mutant studies to be crucial for the regulation of diapause in response to temperature [251]. The transition into migratory forms and behaviors is highly associated with circadian clocks; perhaps the best known example being that of monarch butterfly (*Danaus plexippus*). The initiation of *D. plexippus* migration is regulated by the circadian clock in response to photoperiod, temperature, and host-plant quality reduction [252]; further to this, the path of migration is also maintained by a time-compensated compass mechanism that is reliant on distinct antennae peripheral circadian clocks [253,254]. 

### 4.2. Foraging, Movement, and Temporal Gating

Foraging activity is an important feature of insect behavior, especially among migratory or otherwise mobile insects and during times of food scarcity. Foraging behaviors are widely acknowledged as rhythmic [255,256], and are particularly plastic, facilitating adaptation to variations in food quality or availability [257]. In many insects, such as Lepidoptera, foraging is a behavior seen in adults, which are no longer herbivorous but rather act as pollinators. However, since oviposition often occurs near food sources, the location of foraging can impact the location of herbivory over time. Importantly, there are also herbivorous insects that forage for plant tissues, such as weevils, leaf beetles, or grasshoppers, and any rhythms in foraging they may exhibit will impact herbivory similarly to a feeding rhythm. Another context in which foraging rhythms become relevant to herbivory outcomes is that of natural enemies, which exhibit rhythms in emergence and use HIPVs to locate their prey [14,220,258,259].

One of the proposed mechanisms by which foraging rhythms could be regulated is the temporal gating of olfactory and gustatory perception. In the model insect *D. melanogaster*, taste sensitivity changes throughout the day, peaking in the morning [260]. Reportedly, this is due to the circadian control of taste receptors via gustatory receptor neurons, and, if the clock is disrupted, there is a surge in food searching behaviors exhibited by the flies [261]. Tobacco hornworm (*Manduca sexta*) exhibits a rhythmic electrophysiology that aligns with their rhythms in foraging behavior in response to a synthetic scent designed to mimic flowers [255,262]. Furthermore, in *Drosophila melanogaster*, the circadian control of olfactory perception by antennal clocks has been described [263,264,265], while PDFergic clock neurons were found to promote rhythmic sensitivity to food odors and associated foraging via a dopaminergic modulation of inhibitory projection neurons [266]. The circadian control of olfaction is also highly relevant to parasitism and predatory behaviors by natural enemies of insect herbivores. During the night, nocturnal predatory earwig (*Doru luteipes*) is able to consistently distinguish volatiles emitted from damaged or undamaged *Z. mays* plants from one another, but this capacity is entirely lost during the day [259]. Since *D. luteipes* feeds at night, this changing ability to sense the call for recruitment from damaged plants appears to support its feeding behaviors. This is especially relevant to the role of natural enemies as an indirect defense in herbivory, since the plant will often take advantage of behavioral rhythms of parasitoids to recruit them and curb the feeding of insect herbivores [258].

### 4.3. Temporal Patterns in Herbivory

#### 4.3.1. Feeding Rhythms

Circadian feeding patterns have been observed in both larval and adult insects [267,268,269,270,271]. Studies of *Drosophila* feeding rhythms have identified regulation involving peripheral, neuronal, and glial clocks [271,272,273,274]. Historically, it has been difficult to distinguish locomotor rhythms from feeding rhythms when observing insect behavior, so other metrics are adopted as a proxy for rhythmic feeding. One of the more common metrics is the diet consumed in specific intervals, a technique used to unveil the feeding rhythms of cabbage looper (*Trichoplusia ni*), *S. littoralis*, and cotton leafworm (*Spodoptera litura*) larvae. In all cases, feeding rhythms were maintained during constant darkness following entrainment to a photocycle, with more food consumed during the subjective night [15,268,275]. Notably, *T. ni* feeding peaks around the start of the subjective night, *S. litura* in the middle, and *S. littoralis* towards the end. Another example of alternative metrics for feeding behavior is the observation that green peach aphid (*Myzus persicae*) also follows a 24 h feeding rhythm, peaking during the early subjective night, by measuring honeydew droplets excreted by feeding aphids left in 4 h intervals [221]. A wide diversity of feeding assays has been developed in *Drosophila* [276,277] and some of these may also be useful for application in other insects. Observations of rhythmic clock gene expression in the *S. littoralis* midgut suggest that this peripheral clock may influence feeding rhythms [268], which aligns with earlier observations in *Drosophila* implicating peripheral clocks in digestive tissues in the control of rhythmic feeding [271].

#### 4.3.2. Metabolism and Digestion

In addition to feeding often being highly rhythmic, feeding itself is a strong zeitgeber for certain circadian clocks, including different types of food affecting the regulation of clock genes in varying species [278,279,280]. This indicates a significant interaction between the supply of energy and resources and circadian regulation; however, less research on this has been carried out in insects compared with mammals. Rhythmic feeding in insects has been widely shown, as discussed previously; therefore, it is likely that associated metabolic pathways interact significantly with the insect circadian clock. This is supported by the prevalence of peripheral clocks in various insect metabolic organs such as the fat body and Malpighian tubules [268,281,282].

The release of digestive enzymes from these organs is regulated by self-sustaining peripheral clocks that have been shown to be entrained by light [281,283,284], food [32,285,286,287,288], and through interactions with brain clocks [289]. This highlights both the importance and likely fitness benefits of anticipatory digestive enzyme activity and how the correct timing of feeding contributes to metabolic efficiency. Research on *S. litura* showed the timed release of digestive enzymes with significant daily rhythmicity in the expression of detoxification genes in the larval midgut and fat body, peaking during the larval inactive phase [275], potentially highlighting the importance of a rest and digest phase for plant material digestion and detoxification. 

The specific regulation of digestive enzymes in response to feeding is likely a significant contributor to herbivorous larvae’s ability to feed on host plants within and outside host ranges [290]; therefore, the correctly timed release of such would be important for efficient feeding. Signals from food in the gut have been shown to be a key regulator of digestive enzyme release [291]; nevertheless, the endogenous signals for production and release of such can be maintained rhythmically. Amylase release is shown to occur rhythmically, peaking around dawn [268,275], though this is likely strongly associated with food presence in the gut, as shown in two-spotted cricket (*Gryllus bimaculatus*) [292]. However, the neuropeptide adipokinetic hormone (AKH) is a key regulator for amylase and contributes to the regulation of rhythmic behaviors through the management of energy expenditure [293], so there may be further indirect circadian regulation of amylase outside of rhythmic feeding. Various other digestive enzymes were shown to have similar circadian rhythms in activity [268,275], such as trypsin [294] and trehalase [295], with the latter reported to exhibit strong sensitivity to shifts in the photoperiod [296]. The expression of the protein responsible for the synthesis of the trehalase substrate also shows a significant rhythm [268]. The circadian regulation of gut absorption rates and sensitivity to specific molecules has not yet been categorized by research. 

Outside of digestion, the breakdown and conversion of energy stores may help sustain rhythmic activities. AKH from the Corpora Cardiaca acts in analogy to vertebrate glucagon by promoting the release of trehalose and triglycerides via its cognate receptor (AKHR) from the fat body into the hemolymph [297,298,299]. Moreover, AKH mediates both starvation sensitivity as well as starvation-induced hyperactivity, which can override normal clock-dependent behavior [297,298]. The mobilization of lipids and other energy storage molecules such as glycogen occurs rhythmically and can be under circadian clock regulation, timed with species-specific activity windows [271,300,301]. Indeed, metabolic tissues such as the fat body exhibit rhythmic gene expression as a result of both food-entrained tissue-specific circadian clock function as well as more direct food-associated responses [285]. This metabolic timing may be particularly important for the energy demands of flight, allowing for energy availability to be timed with flight initiation, similar to how some lepidopteran species “shiver” to heat up flight muscles [302,303]. This may also be evident by the increased circadian rhythmicity in flying *Gryllus* individuals, which have to be more efficient with energy availability and use due to the increased fitness costs of flight [304].

#### 4.3.3. Detoxification and Chronotoxicity

Insects possess a rhythmic innate immune system that provides a generalist response to potential toxins, typically converting them to non-toxic products or sequestering them to prevent metabolic disruption [305,306]. Part of these systems is the use of metabolizing enzymes to detoxify xenobiotics and oxidative stress caused by the ingestion of plant material. The regulation of these enzymes shows circadian rhythmicity that is lost in clock mutants [23,307,308]. The fat body and midgut excrete a significant quantity of these detoxification enzymes and, as discussed previously, maintain their own peripheral circadian clocks [275,283,285]. *S. litura* has been shown to rhythmically express detoxification genes from these organs, likely in response to the plant material and secondary metabolites in the gut [275], with *B. mori* showing that these rhythms can be maintained independently in gut tissues [283]. Research on Chinese oak tussar moth (*Antheraea pernyi*), however, also showed a reliance on the central circadian clock [289], indicating that the regulation of detoxification rhythms can often be species-specific.

Cytochrome P450s constitute a large family of broadly acting detoxifying enzymes, crucial for insects’ ability to detoxify the variety of secondary metabolites ingested during herbivory [309]. They have been shown to have significant circadian rhythmicity, providing fitness benefits that are lost in clock mutants [23,310]. Changes in the rhythmic expression of numerous other detoxification enzymes have also been recorded in *Clk* mutant *D. melanogaster* [120]. Plants express secondary metabolites rhythmically; therefore, specialist detoxification enzymes such as glucosinolate sulfatases (GSSs), crucial for the detoxification of the mustard oil bomb used by cruciferous plants as an herbivory deterrent, may also be maintained rhythmically to counter plant defenses. “Chronotoxicity” refers to the changes in toxicity as a function of the time of day of exposure. Diel differences in the toxicity of infections or compounds in insects have been well documented, along with the impact of insect feeding and detoxification rhythms on crop losses and pesticide effectiveness [306,311,312,313]. These changes in outcomes are likely a factor of the circadian regulation of innate immunity discussed above. In *D. melanogaster*, many detoxification genes show significant increases in expression after midday, matching a phase of increased resistance to the acute effects of the insecticides propoxur and fipronil [314]. Similar results were found in housefly (*Musca domestica*), showing clear peaks in insecticide susceptibility just prior to midnight [315]. *S. litura* also showed significant rhythms in detoxification genes and insecticide susceptibility, showing significantly higher mortality to dark phase insecticide exposure [275]. Glutathione-s-transferases specifically were shown to be regulated by circadian clocks, maintaining expression rhythms into constant dark conditions, along with rhythmic susceptibility to the insecticide permethrin [316]. These findings show how insects’ abilities to detoxify both natural and synthetic herbivory deterrents are closely tied to circadian clocks and are important to consider for insect herbivory and control [317].

## 5. Temporal Interactions Between Insect Herbivores and Plant Hosts

Some aspects of the temporal control of herbivory are more generally shared across insect–plant systems, while others are unique to the specific species involved. Generic wounding responses, including the induction of JA and ethylene by chewing insects and SA by piercing and sucking insects [318], are gated so that wounding at different times of day elicits greater or lesser responses [215,237], which contributes to the temporal relationship within insect–plant systems. Evolutionary pressures and biological arms races have guided the development of specialized characteristics of herbivory within specific insect–plant systems, evident from the intricate interactions between insect specialists and their chosen hosts, and in the tailored responses of plants to infestation by a specific insect (reviewed by [4]). In this chapter we will describe how insect–plant systems have evolved in the context of the temporal aspects of herbivory, before expanding on what has been discovered in terms of the effects of circadian timing on insect–plant systems using temporal alignment as a tool.

### 5.1. Evolutionary Aspects of Temporal Insect–Plant Interactions

Many insects have evolved responses to the mustard oil bomb in crucifers, ranging from aphids that sequester GSLs to prevent their conversion by myrosinases [319], to detoxification via specifier proteins in cabbage white butterfly (*Pieris rapae*) larvae [320] or GSS in *P. xylostella* larvae [230]. *C. sinensis* produces blends of HIPVs that reflect herbivore species and density, as well as infestation time [321]. Not only the complexity but also the diurnal rhythmicity of these volatile blends varies depending on the species of herbivorous insect. It has been stipulated that the specificity of both the HIPV blend and the rhythm in emission is synchronized with the rhythmic emergence of natural enemies in order to recruit the correct parasitoid at the appropriate time [216,220]. Natural enemies of insect herbivores discriminate minute details between plant infestations via HIPV communication as part of their mutualistic relationship with host plants [258] and often show rhythms in emergence [14]. It follows then that plants may have evolved specific rhythmic interactions with natural enemies of their herbivores over time. 

Another hallmark of the evolution of temporal control within herbivory is the synchronization of plant defenses with insect feeding behaviors, as seen in the relationship between generalist insects and *A. thaliana* [15]. Temporal synchronization in this interaction was thought to benefit the plant and therefore implicates the evolution of this interaction in such a way that the plant can limit its resources temporally to minimize harm. Alignment or misalignment within an herbivory dynamic often determines at least in part the herbivory outcome, but the specific way in which it impacts overall outcomes is frequently specific to the insect–plant system. This may be due to the different pressures experienced by organisms within a specific system, and the subsequent ways they have evolved or adapted to these pressures that ultimately change how they are affected by shifting temporal alignment.

### 5.2. Temporal Alignment of Herbivory Within Specific Insect–Plant Systems

Scientific studies of the impacts of circadian rhythms on insect herbivory have mostly been one sided, focusing on either plant defense mechanisms or insect behaviors. As described above, plants and insects interactively contribute to the rhythmic control of herbivory with environmental factors also playing a role. A more meaningful understanding of circadian plant–insect interactions, therefore, requires studies investigating both sets of organisms simultaneously.

A small but growing number of studies are contributing to our understanding of the temporal control of insect herbivory (see Table 1). One of the hallmarks of interactions between clocks of plants and insects is the synchronization of their behavioral and physiological rhythms. The tri-trophic interaction of *P. lunatus*, pea leaf miner *(Liriomyza huidobrensis*) and the leaf miner parasitoid (*Opius dissitus*) is a clear example. This system is synchronized by light input driving circadian rhythms in pea leaf miner feeding and diurnal HIPV emission rhythms from damaged leaves [14]. Simultaneously, endogenous rhythms in leaf miner parasitoid emergence facilitate the recognition of distinct blends of HIPVs at specific times of day, recruiting them to the plant at the time of peak leaf miner feeding. This synchronization is maintained, albeit weakly, to an extent in constant light conditions. In this system, the behavioral and metabolic rhythms of the plant and the insects are determined by the light phase and influenced by the clock outputs of the other organisms in the system. Light, however, is not the defining factor for temporal regulation in every system. Other interactions that showcase temporal synchronization are those of *Z. mays* and northern armyworm (*Mythimna separata*), which are temporally coordinated by the emission of plant volatile organic compounds (VOCs) [322]. The larvae exhibit rhythmic “hiding” behaviors during the light phase, which can be entrained to a photocycle without the presence of plants, but it was determined that their responses to plant volatiles override the influence of light and re-entrain their clocks to the plant photocycle. Behaviors of northern armyworm larvae fed on an artificial diet and exposed to volatiles from corn plants kept separately consistently aligned with the phase of the photocycle condition that the plants were in. This was true even when larvae and plants were antiphase to one another, suggesting that the synchronization of the northern armyworm behavior to the rhythms of host plant volatiles is favored over light input. In damaged tea plants *C. sinensis*, the tight circadian regulation of HIPV benzyl nitrile emission ensures that the recruitment of natural enemies, including stink bugs (*Eocanthecona furcellata*), is synchronized with the typical feeding rhythms of tea geometrid (*Ectropis obliqua*), while simultaneously repelling tea geometrid larvae and inhibiting their growth [219,220].

The benefits to different organisms of synchrony or asynchrony within insect–plant systems are also beginning to be explored. By shifting the temporal alignment of two organisms, it becomes possible to compare organisms that are “in-phase” (IP) or “out-of-phase” (OP) with one another, where the IP set experience subjective times of day at the same time and the OP set do not. This makes it possible to identify interactions in which the clock of one or both organisms plays a role in herbivory outcomes. Generalist crop pest *T. ni*) larvae fed on OP *A. thaliana* thrived in comparison with larvae fed on IP plants. OP larvae caused significantly more damage to their hosts and ended up significantly larger with greater bodyweight than their IP counterparts [15], suggesting that plant resistance is significantly enhanced by the synchronization of the plant and insect clocks in this system and indicating that the plant clock may indirectly mediate insect herbivory. Arabidopsis mutants with a defective JA defense pathway lost the advantage associated with an IP relationship to the *T. ni* herbivores, leading to the conclusion that rhythmic JA accumulation was responsible for the observed phase-alignment phenotypes. If larvae fed outside of the times anticipated by the plants, they could take advantage of the “lowered” defenses. The idea that plant–insect co-entrainment confers a defensive advantage to the plant was then tested on a wider variety of common crop plants, which led to similar results. As with *Arabidopsis*, feeding on OP cabbages caused more damage and superior larval fitness than IP cabbages [214]. This remained true, to the same extent, for non-Brassica crops, implying that the temporal relationship of cabbage loopers to their hosts is important regardless of the host species. The benefit for the insect of asynchrony between pest and host appears to extend beyond cabbage loopers. *M. persicae* exhibited a significant preference for OP wildtype (Col-0) *A. thaliana*, a phenotype which was lost when aphids were fed on mutants with no robust clocks [221]. While there was no significant difference in bodyweight, the preference was likely related to the timing of indole GSL accumulation relative to the aphid’s feeding rhythm.

In both of these cases, the synchronization of the rhythms within the interaction seems to be driven by the plant clock, with OP entrainment conferring an advantage to the pest, but this is not always so clearly defined. Larvae of the specialist pest *M. sexta* feeding on wild tobacco (*N. attenuata*) did not appear to have a consistent significant advantage in OP alignment [324]. Nevertheless, some *N. attenuata* defense compounds exhibited phase alignment-associated differences in a photocycle-dependent manner, with OP plants producing significantly less nicotine and 17-hydroxygeranyllinalool diterpene glycosides in constant darkness and increased titers of caffeoylputrescine in constant light [212,324,326]. However, this metabolomic data represented a snapshot rather than a profile across the day, making it impossible to assess if and how rhythms in plant defense were impacted. While it is apparent that the impact of circadian phase alignment on herbivory is context dependent, additional studies are needed to explore how its importance differs across different pest and host plant combinations. Interestingly, a study investigating the effects of the plant clock on predation of *M. sexta* eggs on *N. attenuata* plants found that when the plant was lacking a robust clock, predation decreased significantly [325], likely as a result of perturbations in the typical HIPV emission rhythms in response to egg presence. Notably, this was a field study, meaning the specific natural enemies were not identified and may have been from multiple species. Nevertheless, such results in a field setting are encouraging as they are likely more applicable to a real-world plant–insect dynamic.

The mechanisms by which herbivory interactions are dynamically impacted by the clocks of the relevant organisms are varied, complex, and likely unique to specific systems. Notably, in many systems, synchronization between organisms arises as a core concept underpinning their dynamic, which once again highlights the role of evolution in these systems. Understanding how the relative phase of plant and insect clocks and resulting changes to plant defenses or insect behaviors could lead to the characterization of how herbivory can be shaped by the reciprocal influence of plant and insect clock outputs on one another.

## 6. Applications and Future Directions

### 6.1. Implications for Agricultural Pest Management

Chronoculture of crops entails the alignment of agricultural practices with innate circadian and seasonal rhythms [327] and takes advantage of a wide variety of clock-controlled aspects of plant physiology, including, for example, nutrient uptake and herbicide resistance [328,329]. The impact of both plant and insect circadian rhythms on defense and detoxification pathways also provides a strong rationale for taking them into account in chronocultural approaches.

More robust maintenance of plant circadian rhythms, through the maintenance of environmental conditions, may increase resistance to pests post-harvest, improving the quality and quantity of crops available for sale. In-phase photocycle entrainment of postharvest crops within 3 days of harvesting was found to have a protective effect against herbivory in storage for up to 1 week [214].

Harnessing the effects of the plant clock to improve strain survival and defense against general or targeted pests in specific environments should be possible utilizing modern artificial selection and genome editing. Entrainment of the plant clock impacts both fitness and herbivory resistance, as discussed previously [15,330]. Therefore, one approach to improve crop strains might be to select for the upregulation of known defenses with specific daily/annual timing, where there is the greatest risk from pest species or other environmental stressors [331,332,333]. By incorporating the rhythmic nature of herbivory into this approach, i.e., by temporally restricting or appropriately timing the defense upregulation in a plant, it may be possible to circumvent the obvious drawbacks to constitutively increasing plant defenses posed by the growth–defense trade off, such as decreased fertility or biomass accumulation [183,210]. Conditional enhancement of defensive responses might be achieved by genetic improvement, which, for example, results in increased defense levels under particular photoperiods or temperatures. It may also be feasible to create plants with seasonally gated expressions of insecticidal toxins such as *Bacillus thuringiensis* toxin [334]. The harnessing of HIPV regulation to better prepare plants for seasonal changes in pests may help further reduce losses through the defense mechanisms previously discussed. Applications of artificial HIPV, or HIPV supporting fertilizers, in response to acute pest pressure can also be an additional tool that growers can use to respond to specific pests.

Insect circadian clocks offer many avenues for increasing treatment efficacy from insecticides to integrated pest management strategies. Temporally optimized trapping has the potential to improve the recording and tracking of pest species through timed pheromone and bait releases when the targeted pests would be most sensitive, reducing costs and or lifespan of traps, equally making them more effective for direct crop protection [335,336,337]. The application of both broad spectrum and targeted insecticides will also benefit from exploiting rhythms in pest susceptibility. Understanding diel changes in exposure risk to chemical controls may be crucial for improving the efficacy of chemical treatments and improving cost/benefit ratios for farmers. For example *S. litura*, as discussed previously, shows highly rhythmic feeding and detoxification, feeding during the dark while hiding and burrowing at soil level during the day, with significantly increased detoxification rates [268,275]. Pesticide application could also be temporally aligned with insect rhythms in oviposition, emergence [338,339], or ecdysis [340,341] to target ovipositing adults or newly emerged adults or larvae that have just molted.

Periods of increased susceptibility can also be exploited with the help of natural enemies. It could be important to consider circadian clocks when choosing natural enemies for specific pests. Species used as biological control agents may be more effective if their circadian rhythms align with those of the target pest, increasing the exposure between the pest and its natural enemy. Initial releases of these may also benefit from this consideration by being timed to coincide with periods of pest activity, such as oviposition, or with periods of increased pest vulnerability, such as during ecdysis. Timing the release of sterile insect technique (SIT) populations may also greatly benefit their efficacy. As the greatest survival rate and dispersion of SIT populations occurs following release [342,343,344,345], exploiting peaks in target pest species activity, including nearing or during reproductive activity peaks, may maximize exposure and breeding between wild and SIT populations. 

### 6.2. Climate Change Impacts on Temporal Insect–Plant Interactions

It may be tempting to assume that the well-studied direct impacts of climate change, such as drought or heat stress, can be used in isolation to reliably predict how climate change will alter relationships between plants and insects. While this approach provides vital information that can be used to understand the impact of temperature or humidity on individual interactions, it fails to account for the nuance and complexity of the changing environment that the interaction is embedded within. In reality, the effects of climate change involve an array of direct and indirect interactions within ecosystems that may be less straightforward to predict.

The combination of multiple plant stressors can disrupt plant homeostasis [346]. Moreover, it can impact circadian functioning. One of the stressors that many plants will face in the coming years is drought stress, which has the capacity to modulate both the phasing and expression of core oscillator genes in *A. thaliana* and soybean (*Glycine max*) [347,348]. This will undoubtedly have detrimental knock-on effects to the correct phasing of direct and indirect plant defense, especially for *A. thaliana*, a plant that is known to benefit significantly from the synchronization and appropriate phasing of its jasmonate and salicylate production [15,221]. Drought stress induced an advanced phase for *G. max* homologs for TOC1, LUX, and PRR7 [347]. As previously discussed, LUX is thought to play a role in the temporal regulation of JA biosynthesis, which in turn acts a master regulator of defense against biotic stressors [223], so incorrect *GmLUX* phasing brought on by climate change-induced water stress could weaken the defenses employed by *G. max* during herbivory. Ultimately, drought stress-induced altered phasing of the core clock has the potential to significantly affect herbivory outcomes, in part via JA biosynthesis regulation, and may be associated with increased damage. Notably, not all regions will experience water stress, as climate models predict varying changes to precipitation in different regions within the global atmospheric circulation system in the coming years [349].

The role of circadian clocks in mediating the impacts of climate change is most evident in the adjustment of seasonal rhythms. One of the expected changes of global warming is that areas may be warmer than anticipated based on temperatures historically associated with a particular time of year. Climate change, thus, brings about shifts in the relationship between day length and temperature, which both serve as seasonal timing cues that can be read by the circadian clocks of plants and insects. Many of the behavioral and physiological outputs that mediate insect–plant relationships are seasonal, such as flowering [350] or diapause and migration, and their regulation is controlled via the integration of both day length and temperature [347,348]. As previously discussed, temperature-dependent diapause regulation (induction and termination) is mediated via the core clock in many insects, as with photoperiod-dependent diapause regulation. Core clock genes in *B. mori*, including *per*, *tim*, *Clk*, *cyc*, and *cry2* act upstream of diapause hormone signaling pathways and cerebral GABAergic pathways to regulate diapause in a temperature dependent manner [251]. This is particularly relevant to insect–plant systems that involve more than one insect, e.g., in multitrophic interactions that include natural enemies. Multitrophic systems of plants, insect herbivores, and their natural enemies are fine-tuned to a very specific temporal dynamic that can be toppled by environmental changes [14]. Moreover, if the organisms within these systems exhibit different responses or adaptations to climate change, for example entering diapause or migrating at different times, the shift in the day length–temperature relationship can disrupt the seasonal restrictions on peak herbivory, as well as interactions between insects within the same system. The dynamic within an insect herbivore–natural enemy relationship is in part mediated by the degree of temporal and spatial overlap they experience [351]. This means that if they are active at different times of year their relationship may change. The outcomes of herbivory, including the extent and timing of damage, will change proportionately to changes to the temporal and spatial overlap of different insects’ activity that act in the same system. These changes will be unique to each interaction as they each contain unique combinations of insects, all of which have the capacity to respond differently to climate change in relation to how they overwinter.

### 6.3. Knowledge Gaps and Research Opportunities

While a wealth of data underscore the roles for both the plant and insect clocks in determining the nature and extent of both herbivory and defense, the precise mechanisms by which this occurs have not been conclusively defined for any insect–plant system.

The identification of various core clock transcription factor binding sites on the promoters of genes essential to defense compound synthesis, underpinning the argument that there may be a direct link between the clock and these pathways [219,221]. What remains to be assessed is whether the circadian regulation observed is the result of direct interaction between the core plant clock and these promoters or if it is an indirect effect of regulation upstream, via gating of phytohormone signal transduction or even further removed via rhythms in JA, ethylene, or SA biosynthesis. Relating to this, a capacity for direct transcriptional regulation by a core clock protein has been proposed for a handful of regulator genes such as MYC2/3/4, relating directly to defense compound biosynthesis. That being said, the impact of the circadian regulation of MYC2/3/4 expression on plant defense rhythms remains to be verified. Confirmation of these would require thorough investigations in vivo assessing the binding of clock transcription factors to defense pathway gene promoters (e.g., using ChIP) and replacing known circadian-regulated transcription factors upstream of defense pathways with non-cycling mimics. 

Recent work has also uncovered that insect feeding behaviors can impact the regulation of gene expression in plants to a greater extent than just the induction of defense. In the interaction between durum wheat (*Triticum turgidum* ssp. *durum*) and bird cherry-oat aphids *(Rhopalosiphum padi*), the onset of aphid feeding is associated with the loss of rhythmicity in the expression of ~5600 rhythmic genes, while ~5200 genes become rhythmic [323]. The mechanism by which this is controlled has not yet been determined, but it was suggested that it could be in response to the rhythmic expression of salivatory proteins, including RpC002, which is essential for continuous feeding in *R. padi*. Genes that gained rhythmicity were enriched for plant defense and WRKY transcription factors, the latter of which is known to feed back onto SA biosynthesis. WRKY28 and WRKY45 upregulate ICS1 and PBS3, respectively [352], both of which are necessary for the biosynthesis of SA via the isochorismate pathway in *Arabidopsis* (Figure 3). The switch to rhythmicity of a large number of genes is such a dramatic effect that it necessitates further study, and unveils additional, previously untouched layers of complexity to how clocks regulate and are regulated by herbivory. It would be beneficial to understand what the real-world outcomes of this switch to rhythmicity/arrhythmicity are in the context of the success of the aphid and the plant within this system, and whether there is a functional difference between plants that can alter the rhythmicity of their gene expression in response to aphid infestation and those that cannot.

Another research priority is the study of the impacts of timed environmental defense cures on herbivory outcomes. As discussed above, a substantial number of investigations have linked plant clock manipulations or mechanical wounding to defense metabolite production in plants; expanding on this could entail studying how actual herbivory of appropriate generalist or specialist insects is affected by such perturbations. Similar studies could also be conducted to assess the defense-enhancing effect of pre-treating host plants with relevant HIPV components. Moreover, it will be important to explore how herbivory outcomes are shaped by the insect and the plant species involved by conducting comparative studies that, for example, pair a host plant with a generalist versus a specialist pest. As discussed above, manipulation of plant–insect phase relationships has been shown to provide valuable insight to the role of their clocks in shaping their interactions [353]. Such studies could be coupled with more detailed transcriptomic and metabolomic analyses that might facilitate the selection of candidate genes/metabolites for further study. It may also be beneficial to repeat prior experiments in which stressor timing was varied, with subsequent omics analyses conducted over a time-series to establish the functional role of clock gating more firmly. 

## 7. Conclusions

**Figure 4:**
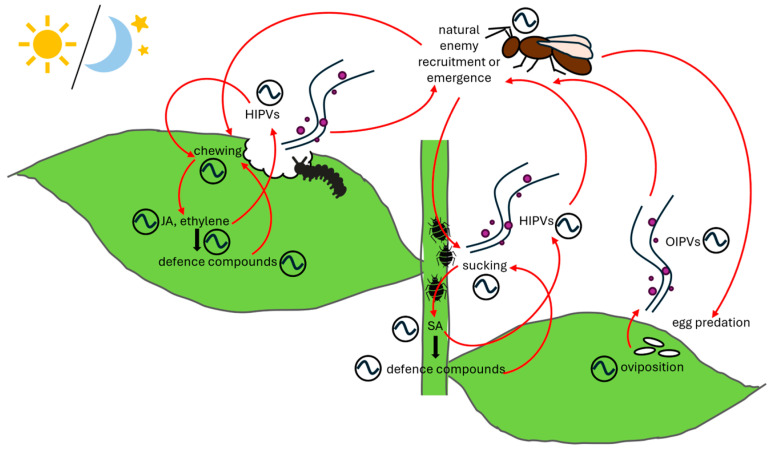
Circadian rhythms in tri-trophic interactions. The defining features of tri-trophic interactions in insect herbivory are primarily rhythmic, and feedback onto one another to maintain synchrony and balance [14]. Circled sine waves denotes circadian rhythmicity, red arrows indicate feedback from one clock output to another and volatile cues are indicated by purple circles and wavy lines.

In this review, we have described how insect behaviors, many of which are highly relevant to herbivory, and plant defenses against chewing and sucking insects are under either direct or indirect circadian control. The phasing of these clock outputs, aligned with zeitgebers like light and temperature, feedback reciprocally onto one another, allowing insect–plant systems to reach a state in which the rhythmicity of each organism impacts and influences the others (Figure 4). For some generalist pests, phase alignment between plant defense metabolite accumulation and insect feeding directly affect herbivory outcomes. In other systems, relationships between insect herbivores and their natural enemies can be defined temporally by rhythmic communication from the plant, via emission of HIPVs or OIPVs.

Across these insect–plant associations, circadian rhythms in both organisms exhibit bidirectional regulatory interactions that shape the dynamics and outcomes of their ecological interplay (Figure 4).

Finally, we have highlighted the necessity for future research to take into account how organisms interacting with one another can influence each other’s gene expression, metabolism, or behavior, and demonstrate thereby that it is necessary to take into account the circadian rhythm in these relationships. A deeper understanding of the temporal regulation of herbivory will not only help unveil the underpinning complex multi-trophic interactions between insects and plants but also illustrate how these contribute to balance plant defense versus plant growth.

Integrated pest management solutions that implement timed rather than constitutive enhancement of the defensive capacities of crop plants may help avoid trade-offs in biomass accumulation or fertility. Incorporating chronocultural practices to increase efficiency and safety of pest control, by taking into account the circadian rhythms of all organisms in the interaction to reduce off-target effects, will be vital to reduce reliance on pesticides and promote sustainable practices in agriculture and forestry.

## Figures and Tables

**Table 1:** Studies that have identified temporal complexity relevant to determining herbivory outcomes.

Class	Insect(s)	Plant Host	Interaction	Relevance	Reference
Generalist insect herbivore	Green peach aphid (*Myzus persicae*)	*Arabidopsis thaliana*	Aphids preferentially settle on out-of-phase wildtype plants; this phenotype is lost in clock mutant lines	The *A. thaliana* clock or its outputs are a determinant in aphid feeding behaviors	Lei et al. (2019) Plant Physiol. [221]
Generalist insect herbivore	Cabbage looper (*Trichoplusia ni*)	*Arabidopsis thaliana*	Larvae fed on out-of-phase plants have a greater body mass, and cause more damage, than those fed on in-phase plants	The plant clock or its outputs are a determinant not only in *T. ni* larvae feeding behaviors but also in herbivory outcomes for the caterpillar	Goodspeed et al. (2012) PNAS [15]
		Various crops			Goodspeed et al. (2013) Curr. Biol. [214]
Generalist insect herbivore	Bird cherry-oat aphid (*Rhopalosiphum padi*)	Durum wheat (*Triticum turgidum* ssp. *durum*)	Aphid feeding caused plant defense genes and WRKY transcription factors to go from constitutively expressed to rhythmically expressed	Synchronization of plant defenses with rhythmic salivary effector production associated with aphid feeding may benefit the plant, as in other examples	Goldstein et al. (2025) BMC Plant Biol. [323]
Generalist insect herbivore	Cabbage moth (*Mamestra brassicae*)	Broccoli (*Brassica oleracea*)	Plants pre-wounded at ZT0 were damaged significantly less by *M. brassicae* over a 24 h period than plants pre-wounded at ZT4 or ZT8	The time of day of plant wounding, possibly through an altered accumulation of metabolites, can modify the behavior of the insect	Doghri et al. (2022) Front. Plant Sci. [215]
Specialist insect herbivore	Northern armyworm (*Mythimna separata*)	Corn (*Zea mays*)	VOCs emitted by *Z. mays* during the light or dark phase were pumped into a *M. separata* larvae enclosure; larvae exhibited classic hiding behaviors dependent on the plant VOCs rather than their immediate environment	Endogenous *M. separata* larval behavioral rhythms can be overwritten by the circadian phase of the host plant	Shiojiri, Ozawa and Takabayashi (2006) PLoS Biol. [322]
Specialist insect herbivore	Tobacco hornworm (*Manduca sexta*)	Wild tobacco (*Nicotiana attenuata*)	Feeding on clock-shifted host plants was no different than on co-entrained host plants, with the exception of significantly greater larval mass at 7 days of out-of-phase feeding	There is no one size fits all application of circadian rhythms to agriculture as phase alignment did not impact herbivory by this specialist insect pest	Herden et al. (2016) J. Intgr. Plant Biol. [324]
Generalist insect herbivore Specialist natural enemy	Pea leaf miner (*Liriomyza huidobrensis*) Leaf miner parasitoid (*Opius dissitus*)	Lima bean (*Phaseolus vulgaris*)	The tri-trophic interaction between all three organisms is synchronized through their respective clocks and clock outputs	Behavior and physiology of each member of this interaction can impact the others through their various reciprocal relationships	Zhang et al. (2010) PLoS One [14]
Specialist insect herbivore Generalist natural enemy	Tea geometrid (*Ectropis grisescens*) Stink bugs (*Eocanthecona furcellata*)	Tea (*Camellia sinensis*)	Rhythmic benzyl nitrile emission recruits natural enemy *E. furcellata* at times of *E. grisescens* feeding	The *C. sinensis* clock mediates the temporal relationship between *E. grisescens* and *E. furcellata*, affecting the outcome of herbivory and the parasitoid’s success	Qian et al. (2023) Plant Cell Environ. [219] Qian et al. (2024) J. Agric. Food Chem. [220]
Specialist insect herbivore Undefined natural enemies	Tobacco hornworm (*Manduca sexta*) Various natural enemies (field study: species not identified)	Wild tobacco (*Nicotiana attenuata*)	Lower egg predation by natural enemies on plants without a robust clock	The plant clock or its outputs may mediate the relationship between natural enemies and herbivore eggs, not just temporally but in terms of the extent of damage	Joo et al. (2019) J. Intgr. Plant Biol. [325]

## Data Availability

No new data were created or analyzed in this study. Data sharing is not applicable to this article.

## Abbreviations

The following abbreviations are used in this manuscript:

AKH	Adipokinetic hormone
CA1P	2-carboxy-D-arabinitol 1-phosphate
CAM	Crassulacean acid metabolism
CCA1	CIRCADIAN CLOCK-ASSOCIATED 1
CLK	CLOCK
CWO	CLOCKWORK ORANGE
CYC	CYCLE
DBT	DOUBLETIME
dCRY	*Drosophila*-type CRYPTOCHROME
ELF3/4	EARLY-FLOWERING ¾
ETC	Electron transport chain
GI	GIGANTEA
GSL	Glucosinolate
GSS	Glucosinolate sulfatase
HAMP	Herbivore-associated molecular patterns
HIPV	Herbivory-induced plant volatiles
HSP90	HEAT SHOCK PROTEIN 90
IP	In-phase
JA	Jasmonic acid
JAZ	JA ZIM DOMAIN
JET	JETLAG
LBS	LUX binding site
LHY	LATE ELONGATED HYPOCOTYL
LNK1/2	NIGHT LIGHT-INDUCIBLE AND CLOCK REGULATED
LUX/PCL1	LUX ARRHYTHMO; otherwise PHYTOCLOCK1
LWD1	LIGHT-REGULATED WD1
mCRY	Mammalian-type CRYPTOCHROME
OP	Out-of-phase
PDP1	PAR-DOMAIN PROTEIN 1
PER	PERIOD
PHYB	PHYTOCHROME B
PIF3	PHYTOCHROME INTERACTING FACTOR 3
PPC	Phosphoenolpyruvate carboxylase
PPCK	PPC kinase
PRR5/7/9	PSEUDO RESPONSE REGULATOR 5/7/9
RuBisCO	Ribulose-1,5-bisphosphate carboxylase/oxygenase
RuBP	Ribulose-1,5-bisphosphate
RVE4/6/8	REVEILLE 4/6/8
SA	Salicylic acid
SLMB	SUPERNUMERARY LIMBS
TCA	Tricarboxylic acid
TCP20/22	TEOSINTE BRANCHED 1-CYCLOIDEA-PCF20/22
TIC	TIME FOR COFFEE
TIM	TIMELESS
TOC1	TIMING OF CAB 1
TTFL	Transcriptional translational feedback loop
VOC	Volatile organic compound
VRI	VRILLE
ZT	Zeitgeber time
ZTL	ZEITLUPE
